# The Role of AI in Characterizing the DCM Phenotype

**DOI:** 10.3389/fcvm.2021.787614

**Published:** 2021-12-21

**Authors:** Clint Asher, Esther Puyol-Antón, Maleeha Rizvi, Bram Ruijsink, Amedeo Chiribiri, Reza Razavi, Gerry Carr-White

**Affiliations:** ^1^Department of Cardiovascular Imaging, School of Biomedical Engineering and Imaging Sciences, King's College London, London, United Kingdom; ^2^Department of Cardiology, Guys and St Thomas' NHS Trust, London, United Kingdom; ^3^Division of Heart and Lungs, Department of Cardiology, University Medical Center Utrecht, Utrecht, Netherlands

**Keywords:** dilated cardiomyopathy, cardiac magnetic resonance, late gadolinium enhancement, artificial intelligence, deep learning

## Abstract

Dilated Cardiomyopathy is conventionally defined by left ventricular dilatation and dysfunction in the absence of coronary disease. Emerging evidence suggests many patients remain vulnerable to major adverse outcomes despite clear therapeutic success of modern evidence-based heart failure therapy. In this era of personalized medical care, the conventional assessment of left ventricular ejection fraction falls short in fully predicting evolution and risk of outcomes in this heterogenous group of heart muscle disease, as such, a more refined means of phenotyping this disease appears essential. Cardiac MRI (CMR) is well-placed in this respect, not only for its diagnostic utility, but the wealth of information captured in global and regional function assessment with the addition of unique tissue characterization across different disease states and patient cohorts. Advanced tools are needed to leverage these sensitive metrics and integrate with clinical, genetic and biochemical information for personalized, and more clinically useful characterization of the dilated cardiomyopathy phenotype. Recent advances in artificial intelligence offers the unique opportunity to impact clinical decision making through enhanced precision image-analysis tasks, multi-source extraction of relevant features and seamless integration to enhance understanding, improve diagnosis, and subsequently clinical outcomes. Focusing particularly on deep learning, a subfield of artificial intelligence, that has garnered significant interest in the imaging community, this paper reviews the main developments that could offer more robust disease characterization and risk stratification in the Dilated Cardiomyopathy phenotype. Given its promising utility in the non-invasive assessment of cardiac diseases, we firstly highlight the key applications in CMR, set to enable comprehensive quantitative measures of function beyond the standard of care assessment. Concurrently, we revisit the added value of tissue characterization techniques for risk stratification, showcasing the deep learning platforms that overcome limitations in current clinical workflows and discuss how they could be utilized to better differentiate at-risk subgroups of this phenotype. The final section of this paper is dedicated to the allied clinical applications to imaging, that incorporate artificial intelligence and have harnessed the comprehensive abundance of data from genetics and relevant clinical variables to facilitate better classification and enable enhanced risk prediction for relevant outcomes.

## Introduction

Dilated Cardiomyopathy (DCM) merely describes a dilated and dysfunctional left ventricle (LV) in the absence of significant coronary disease, valvular dysfunction, or poorly controlled hypertension. Documentation of LV size and ejection fraction (EF) are the established measurements by echocardiography or CMR that define phenotype and determine risk stratification. CMR is considered the gold standard, as it provides accurate volume assessment, morphology, function, and tissue characterization all within a single assessment to better describe underlying cardiac pathology.

**Figure 1:**
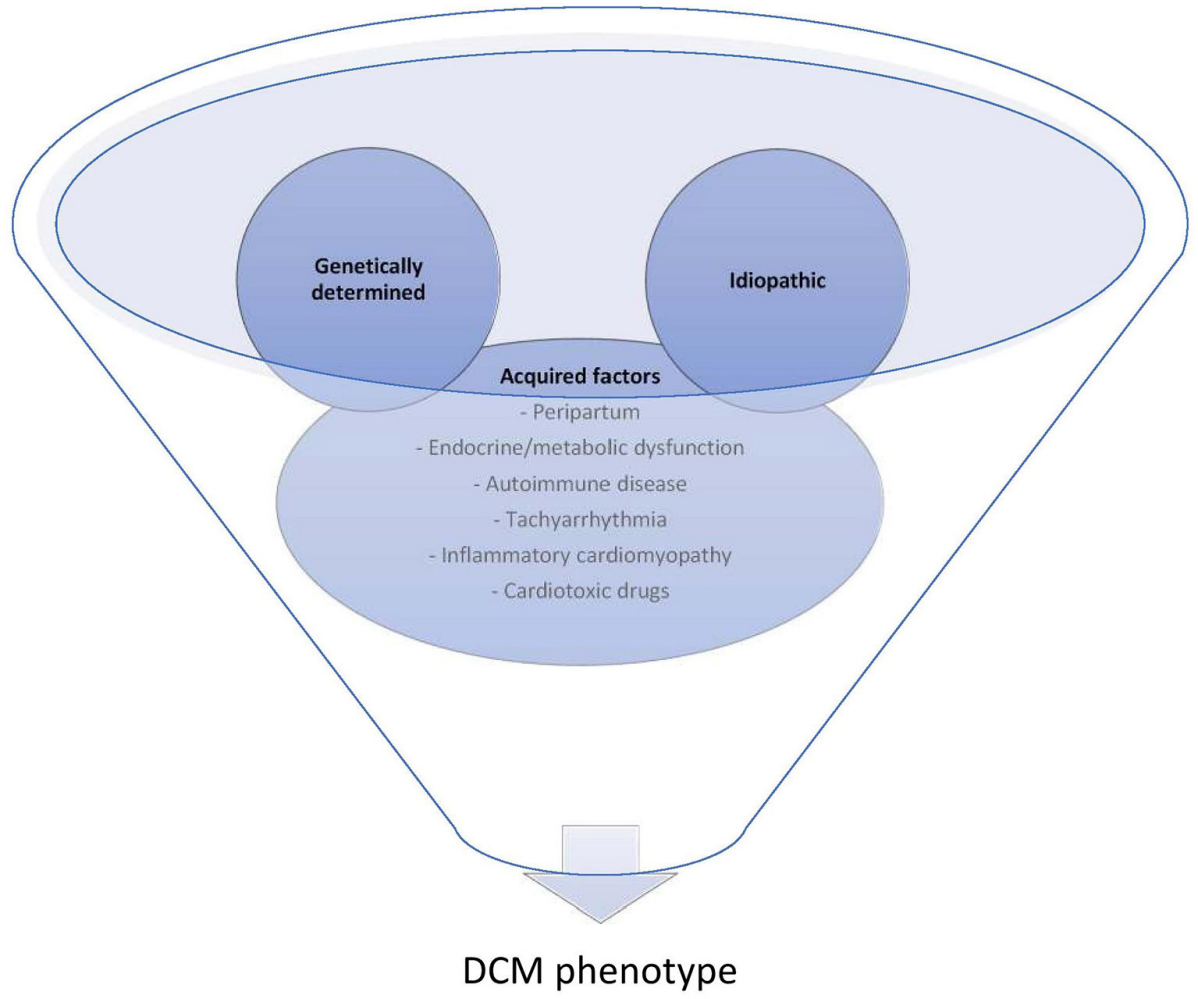
Complex interplay of environment with genetic factors contribute to the DCM phenotype. Commonly overlooked acquired factors that are either reversible factors for those with “idiopathic” DCM or can contribute to the clinical expression or progression of those with underlying genetic predisposition.

It is increasingly appreciated that DCM is not simply the single disease entity of “non-ischemic” heart failure, but rather, represents a unique family of heart muscle diseases with complex interactions between genetic predisposition and environmental precipitants (see Figure 1) (1–3).

As such a clinical spectrum of DCM exists, with variable expression of arrhythmic and functional changes over time (4). Genetic testing clearly provides a fundamental insight into discriminating part of these diverse DCM subtypes; however, the complex interplay of genetics and environmental influences dictates for a deeper characterization of the DCM phenotype through advanced imaging techniques. This would also be warranted in the matter of risk stratification, which to date, remains particularly challenging for this cohort and appears to be inadequate when focused on the single parameter of LVEF (5). Evidently, a non-negligible proportion of DCM patients suffering from sudden cardiac death have much milder reductions in LVEF that do not meet consensus criteria for primary prevention implantable cardioverter-defibrillator (ICD) (6). Furthermore, at least a third of adverse events can occur later in the course of the disease, negating some of the reliability of static, solitary measures of systolic function in predicting long-term outcomes in DCM cohorts (7). There remains a relative lack of robust markers for stratifying patients with the DCM phenotype, and this is highlighted in the DANISH study, suggesting a limited benefit of primary prevention ICD on overall mortality in patients with non-ischaemic heart failure (8). By extracting a multitude of information generated from images and clinical datasets, Artificial Intelligence (AI) potentially holds the essential link to uncovering some of the complex associations between clusters of DCM patients in a fully automated manner. By shifting toward better characterization, it may be ultimately possible to integrate these disease characteristics and multiple novel markers, thereby advancing the refined risk stratification needed in DCM cohorts. This capability does not replace, but rather should augment the clinical decision process in a more efficient, user-friendly way, that hopefully translates into improved patient care.

The rest of the article is organized as follows, firstly, we provide a summary of current methods for diagnosis and characterization of DCM utilizing CMR techniques, followed by recent and key applications of AI within this scope. Subsequently, we highlight the use of AI for risk prediction in DCM and methods that combine imaging and genetic information in DCM characterization. Finally, we provide relevant discussions on current research efforts and future work towards more comprehensive and personalized imaging stratification of this heterogenous phenotype.

## Current Diagnosis and Characterization of DCM

DCM is a heterogenous myocardial disease characterized by several degrees of reduced LVEF. Whilst the majority with this phenotype benefit from outcomes that improve year on year with up to 90% alive and well at 10 years from diagnosis, the natural history remains variable, with often unidentified initiating triggers, and some individuals unfortunate enough to succumb to unheralded life-threatening arrhythmias and sudden cardiac death at the onset of their clinical presentation (9–13).

Understanding the characteristics, evolution and long-term prognosis are key challenges to enabling proper etiological classification, customized surveillance and initiation of appropriate, effective treatment in a timely fashion. Although evaluation in practice rarely deviates from the protocol-driven investigation of heart failure, the heterogenous nature of the disease that directly results in variable clinical and phenotypic expression, dictates for a comprehensive, DCM-focused investigation strategy. Furthermore, risk stratification based on the simplistic evaluation of LV dimensions and LVEF is clearly inadequate across the phenotypic spectrum and our current grasp of suitable predictors of outcomes is still limited.

### The Role of Imaging in DCM

Following detailed history, clinical examination, electrocardiogram (ECG) and laboratory tests that may elucidate features of a specific underlying etiology or secondary organ dysfunction, imaging techniques play a crucial role in confirming the diagnosis, ruling out other competing causes for LV dysfunction, further evaluation of the etiology and in guiding treatment strategies.

Whilst two-dimensional echocardiography is often first line in the diagnostic imaging pathway and has an additional role in both early and follow up function assessment in DCM patients, its role in defining an underlying etiology is limited, particularly with the compromise that occurs in light of inadequate acoustic windows and poor endocardial border definition. Furthermore, due to the inherent geometric assumptions that perform well in healthy individuals with normal sized hearts, volume assessment in those with distorted ventricular size and shape is less reliable, with significant intra- and interobserver variability.

CMR is well-placed in this respect, with unrestricted field of view and high spatial resolution to capture global and regional changes in structure and function irrespective of ventricular geometry or patient habitus (14). As there is less operator dependence for endocardial delineation, the interobserver reproducibility variability for volume and EF quantification is less for CMR than it is in echocardiography (14). This is ideal for both the initial evaluation, where decisions on initiation of medical therapy are based on LVEF thresholds, but also to carefully monitor progression of the disease and the appropriate selection of those who require device implantation. The integration of perfusion and whole heart angiography enables the exclusion of significant coronary disease with a high accuracy, thereby reducing the need for separate ischaemia assessment by computed tomography (CT) or invasive coronary angiography in the initial work up of DCM (15). Thus far, routine use of CMR for diagnosis alone has not been shown to significantly improve the clinical identification of non-ischaemic heart failure causes (16). However, complementary information is offered with tissue characterization and parametric mapping sequences that enable assessment of changes to intrinsic myocardial properties correlating with altered biological pathways. These additional features offer the potential to aid the differentiation of the underlying etiology, enable prognostic assessments and guide treatment options. Although there remains a lack of data from large randomized controlled trials asserting the role of contemporary CMR on impacting patient outcomes, the evolving landscape of techniques and applications for in-depth phenotyping paired with advanced analytics pave an important path toward CMR-guided precision care in the DCM population.

### CMR for Dynamic Cardiac Assessment

**Figure 2:**
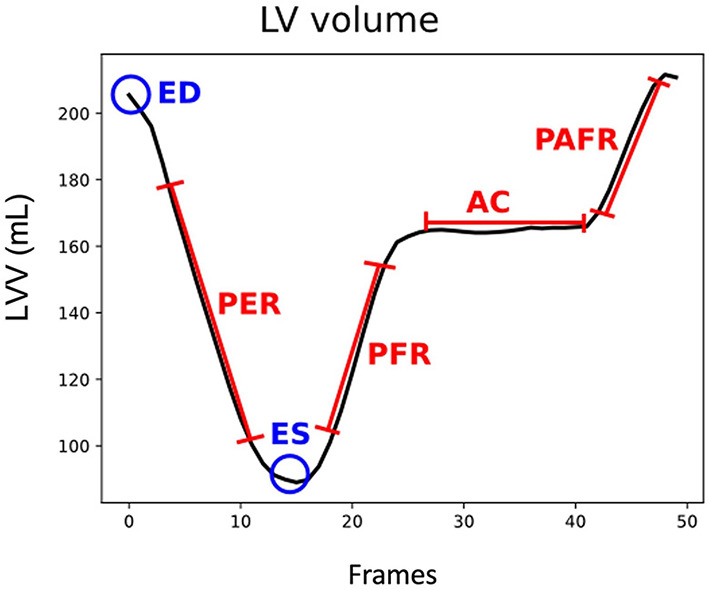
LV volume (LVV) curve for a cardiac cycle, in blue end diastole (ED) and end systole (ES) frames, in red peak ejection rate (PER), peak filling rate (PFR), atrial contribution (AC), and peak atrial filling rate (PAFR) parameters.

Standard CMR provides the gold standard for biventricular volume assessment, further allowing for accurate documentation of systolic function, which is imperative for the investigation of all comers with heart failure. Even though current clinical practice focusses on these static measures obtained from only two end time points of the cardiac cycle, due to real time acquisition over multiple phases, cine-CMR possesses additional information on dynamic volumetric changes. Consequently, it is feasible to generate volume/time profile curves that allow evaluation of continuous ventricular volume changes and extraction of more sensitive parameters of cardiac function such as peak filling rates (see Figure 2) (17). From this, additional indices of filling and ejection are possible to obtain simultaneously, with the potential for more detailed analysis of both systolic and diastolic function (18). Differing LV filling patterns have already been suggested to exist amongst DCM patients with direct implications on the classification of functional status and predicting adverse outcomes (19, 20). However, most studies that assessed these parameters in the DCM phenotype were significantly limited in the diversity of structural and functional heterogeneity seen in most contemporary DCM cohorts, thus hindering full exploration into the evolutions of filling and ejection patterns in different subgroups and their varied clinical outcomes (18, 21). Such studies are warranted but the current tools for obtaining these parameters are limited by the extent of user-interface involved in semiautomatic processing, whereby contours are determined not only at each slice level but also at each time point or phase prior to the computation of volumes needed to produce the curves for each patient.

### CMR Tissue Characterization

Unique tissue characterization sequences add a further dimension to the investigative prowess of CMR in the evaluation of the DCM phenotype. The ability to non-invasively assess and quantitate myocardial tissue properties makes CMR well-suited to unravel the onset and extent of pathogenic processes occurring within the myocardium, that could only previously be determined through high-risk invasive cardiac biopsy.

#### Late Gadolinium Enhancement- CMR

**Figure 3:**
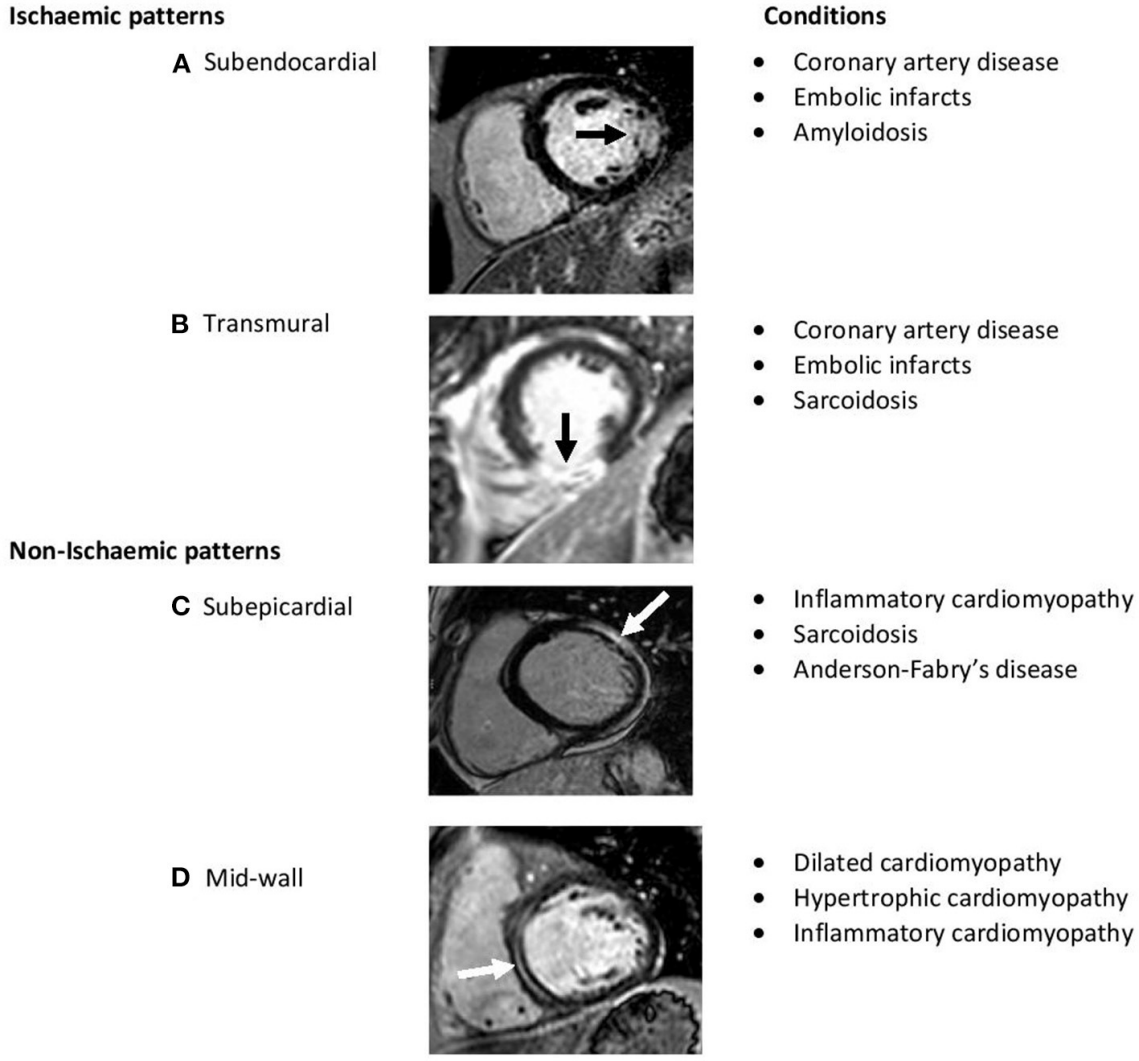
Short-axis late-gadolinium-enhanced CMR images demonstrating hyperenhancement (arrows) indicative of scar. The differing patterns help characterize various myocardial diseases. **(A,B)** Represent typical ischaemic scar pattens involving subendocardium. **(C,D)** Represent non-ischaemic scar patterns which typically involve epicardium to mid wall.

Tissue characterization using the late gadolinium enhancement (LGE)-CMR technique enables the identification and quantification of regional areas of replacement fibrosis; this refers histologically to a process of reparative microscopic scarring occurring in response to myocyte necrosis (21). It has been found to be a clinically useful tool for distinguishing DCM from other important differentials of LV dysfunction such as coronary disease or sarcoidosis, subtyping the etiology of DCM, as well as for predicting the trajectory of the disease (Figure 3) (22, 23). Up to 45% of DCM patients are affected, usually in a mid-wall distribution, with <15% showing an ischaemic pattern that crucially, would not be sufficient to explain the degree of ventricular dysfunction (22, 24).

There is substantial clinical evidence that the presence of fibrosis and its detection *via* LGE-CMR heralds a strong and independent predictor of adverse outcomes in patients with non-ischaemic cardiomyopathy even in the absence of heart failure symptoms (24–32). This is a powerful parameter in the era of personalized risk stratification, especially when current criteria for prophylactic ICD implantation on the basis of significant LV dysfunction has low sensitivity for identifying some high risk patients whose clinical outcomes are not consistently related to LVEF (5).

The identification and extent of LGE at an early stage of the cardiomyopathic disease provides additional information beyond LVEF, thus enabling earlier prognostic characterization and drawing attention to those who might benefit from closer surveillance or earlier consideration of advanced therapies (25, 30). In the study by Gulati et al. (25), mid-wall fibrosis detected by LGE-CMR imaging in a longitudinal study of 472 patients with DCM, was incrementally associated with all-cause mortality and cardiovascular death or transplantation across the entire range of LVEF. In another study of 150 patients, up to 30% with the mutation *PLN* (phospholamban) p.Arg14del had LGE on CMR with a normal LVEF, suggesting this to be an early feature and higher risk of arrhythmias in carriers of this mutation, but also attesting to the phenotypic insights CMR offers for those with underlying genetic substrate (33).

The identification of LGE in clinical practice and certainly demonstrated in the majority of studies evaluating LGE-CMR in DCM, occurs mostly by visual analysis which is clearly subject to inter-observer variability (24). Elucidating the extent of LGE is apparently complementary to detecting its presence in terms of the additional risk stratification beyond conventional criteria. Neilan et al. (31) assessed the extent of LGE using quantitative methods in 162 patients with non-ischaemic cardiomyopathy and assessed for the annual major adverse cardiac events (MACE), including cardiovascular death and appropriate ICD therapy. Over a follow up period for a mean of 29 ± 18 months, quantified LGE extent demonstrated the strongest predictor of MACE over age, sex and LVEF in multivariate analyses with an adjusted HR 7.61, *p* < 0.0001.

Although quantitative methods might provide more consistent validation for the presence of LGE and a measure of the extent of fibrosis, there are also a number of practical limitations; these include the lack of universal access to quantitative software packages, variable extent of fibrosis quantified by different methods and dependence on supplementary, time-consuming contouring of LGE areas (34, 35). Moreover, LGE which relies on differences in signal intensity between healthy myocardium and focal fibrotic areas, appears to be limited in its ability to assess and quantitate diffuse (non-focal) myocardial injury and interstitial fibrosis (36, 37). From a technical perspective, LGE is also affected by inconsistencies in acquisition parameters, such as choice inversion time (TI), and in post-processing when signal intensity thresholds may be arbitrarily applied to distinguish normal myocardium from fibrotic tissue. Finally, despite the strong prognostic value in identifying high risk patients, randomized controlled trials evaluating LGE-based risk stratification are still warranted prior to any guideline recommendation on its use in managing non-ischaemic heart failure cohorts.

#### T1 Mapping

Refined methods in quantitative assessment of tissue characteristics enable routine measurement of diffuse fibrosis, without the reliance on regional differences in tissue contrast intensity (38). Novel techniques comprising of native (non-contrast) and contrast-enhanced T1 mapping represent advances in CMR that enable detection of pathological changes occurring within myocytes and the interstitium in a number of disease states (38). Native T1 is additionally helpful in those unable to have contrast due to contraindications such as pregnancy or severe renal failure. The acquisition of relaxation times during the same cardiac phase enables T1 values to be displayed as a pixelwise map, which can be used to directly quantify myocardial T1 values globally and at specific regions. As this process is not reliant on tissue contrast differences, T1 mapping overcomes the limitations of LGE imaging in detecting diffusely diseased myocardium but has the potential to detect and track myocardial structural alterations throughout the clinical course of disease expression (39, 40).

In DCM, the feasibility of T1 mapping as a surrogate of diffuse fibrosis has been demonstrated at different stages of the clinical phenotype, suggesting a potential biomarker role for non-hazardous follow up in the progression of different DCM cohorts (36, 41–43). This notion is further upheld in the multicenter study of over 600 DCM patients, where T1 indices both regionally and globally showed significant predictive associations with all-cause mortality and likelihood of heart failure-related mortality or hospitalization over a median follow up of 22 months, *p* < 0.001 (44). In a recent study of DCM patients affected by complex ventricular arrhythmias, events thought to be attributable to pathologic remodeling and the inter-related process of diffuse fibrosis, global native T1 time was found to be independently associated with ventricular arrhythmias even after adjustment for LVEF and scar on LGE-imaging (odds ratio 1.14, 95% confidence interval 1.03–1.25; *p* = 0.008) (45). Whilst these studies demonstrate the incremental value T1 mapping may provide in the evaluation of DCM, substantial overlap in T1 values is apparent between those with adverse outcomes and those without (44–46). Accounting for this precise continuum of T1 values with pixel-to-pixel mapping may more reliably differentiate higher and lower risk groups of patients but would be technically difficult and laborious with current manual techniques.

Pre- and post-contrast T1 mapping can also be adjusted for haematocrit, i.e., correcting for the blood volume of distribution, and this introduces an additional technique known as the extracellular volume fraction (ECV), for more focused examination of alterations occurring specifically within the extracellular interstitial compartments (40). ECV appears to have direct relationship with the extent of diffuse fibrosis with good correlation to histopathological quantification and therefore offers a non-invasive, quantifiable assessment of interstitial disease that shows significant promise in prediction of heart failure related outcomes in DCM patients (36, 44, 47). Currently, ECV still has a limited role in differentiating DCM from other causes of non-ischaemic heart failure, due to the significant overlap of values seen across various myocardial diseases (48). However, its particular advantage appears to lie in its reduced sensitivity to variation in scanner field strength, which lends itself applicable toward multi-center and vendor evaluations to assess the extent of its usefulness over LGE-CMR in future DCM studies (47).

#### T2 and T2^*^ Mapping

T2 weighted sequences exploit the biological parameter of T2 relaxation times associated with tissue water content. As such, T2 images and subsequently quantitative T2 mapping can be used for the assessment of myocardial oedema, adding to the aetiological evaluation of active myocardial inflammation as occurs in acute myocarditis (49). The clinical application of T2 mapping to provide additional diagnostic information in distinguishing DCM from healthy myocardium, with the former showing larger and more progressive myocardial water content, was recently supported in a meta-analysis (standardized mean difference 1.90, *p* < 0.01) (50, 51). This could have a pivotal role in the evaluation and differentiation of those who have the functional appearance of DCM due to athletic training from those with pathological myocardial disease (52). However, differentiation of DCM from other forms of non-ischaemic cardiomyopathy is limited in this respect, due to similar changes in T2 values, and due to differences in the sequence acquisition these values may vary from center to center (50, 53). Further research is needed in regards to standardization, verification of its usefulness and timing in the diagnostic pathway, and to better understand the pathophysiological basis for an increase in T2 values in DCM without preceding myocarditis.

T2^*^(star) relaxation mapping is a parameter that shortens due to the local magnetic field homogeneity that occurs with progressive iron deposition. This is useful for the assessment and quantification of iron loading within the myocardium, which can occasionally be associated with a DCM-like phenotype (54). It is a clinically validated tool, with better predictive capability than serum iron biochemistry and can detect the effects of myocardial iron loading earlier than conventional cardiac function assessments (54, 55). As a result, rapid hematological diagnostic pathways can be primed without delay and the response to treatment serially monitored non-invasively using this tool (55).

### CMR for Prognostication in DCM

CMR can confirm and reproduce the assessment of LV mass, volumes, and LVEF, all of which are important indicators for a worse prognosis in severe DCM and other causes of heart failure; the latter two markers being key targets for reverse remodeling and myocardial recovery (56–59). The main limitation of these measures for predictive outcomes is that they are often assessed at initial evaluation, failing to account for the dynamic nature of the disease with favorable response to therapy for a significant proportion of patients; concurrently, they are less sensitive for those with mild-moderate dysfunction who are still prone to significant risk of sudden cardiac death (25, 26, 60).

Risk stratification in this setting is difficult and the current focus of this has shifted toward a multiparametric, dynamic approach, which attempts to incorporate potential biomarkers from biochemistry, ECG signals and imaging (12).

There is increasing evidence for applications within CMR to guide prognostication and subsequent clinical management in DCM. Whilst the majority of these applications for risk prediction are captured through routine assessment, the additional tools, and longitudinal follow-up capability is still regarded as an investigational field of interest within the setting of CMR (14). These current and potential clinical CMR applications in the risk assessment of DCM are outlined in Table 1.

**Table 1:** The current and potential clinical CMR applications for predictive outcomes in DCM.

**CMR Biomarker**	**Current use**	**Studies supporting biomarker for prognostication in DCM cohorts**	**No. of patients studied**	**Median F/U**	**HR/OR for primary end point (95% CI) p <0.05**
LV volume and LVEF	Clinical use	Masci et al. (30)	125	1.2 years	Primary endpoint = CV death and HF hospitalization. LVEDVi HR 1.02 (1.00–1.03), LVEF HR 0.94 (0.90–0.99).
		Gulati et al. (25)	472	5.3 years	Primary endpoint = ACM, cardiac transplantation. LVEF per 1% HR 0.95 (0.93–0.96). LV-EDV index per 10 ml/m^2^ HR 1.09 (1.05–1.13), LVMi per 10 g/m^2^ 1.12 (1.04–1.19).
		Masci et al. (26)	228	1.9 years	Primary endpoint = CV death, congestive heart failure, aborted SCD. LVEDVi HR 1.008(1.000–1.016), LVEF HR 0.962 (0.934–0.990), LVMi HR 1.018 (1.006–1.030).
		Buss et al. (60)	210	5.3 years	Primary endpoint = aborted SCD, CV death, cardiac transplantation. LVEDi HR 1.02 (1.01–1.03), LVEF HR 0.91 (0.88–0.94), LVMi HR 1.11 (1.04–1.18).
RV volume and RVEF	Clinical use	Alpendurada et al. (61)	60	2.2 years	Primary endpoint = ACM, CV hospitalization. RVEF HR 0.96 (0.94–0.99) TAPSE HR 0.88 (0.80–0.96).
		Gulati et al. (62)	250	6.8 years	Primary endpoint = ACM, cardiac transplantation. RVEDVi per 10 ml/m^2^ HR 1.14 (1.05–1.25), RVEF HR 0.95 (0.93–0.97).
		Becker et al. (63)	168	2.2 years	Primary endpoint = ACM, cardiac transplantation, sustained ventricular arrhythmia, appropriate ICD therapy. RVEF per 10% HR 0.74 (0.57–0.95).
LA volume and dimension	Clinical use	Gulati et al. (64)	483	5.3 years	Primary endpoint = ACM or cardiac transplantation. LAVi per 10 ml/m^2^ HR 1.08 (1.01–1.15).
LGE	Clinical use	Assomull et al. (28)	101	1.8 years	Primary endpoint = ACM, hospitalisations for CV event. LGE HR 3.4 (1.4–8.7).
		Cho et al. (32)	79	1.6 years	Primary endpoint = rehospitalisation, cardiac transplantation or death. LGE HR 8.06 (1.03–63.41).
		Masci et al. (30)	125	1.2 years	Primary endpoint = CV death and HF hospitalization. LGE HR 3.96 (1.53–10.3).
		Leyva et al. (27)	97	2.8 years	Primary endpoint = CV death and transplantation. LGE HR 22.0 (4.73–102).
		Neilan et al. (31)	162	2.4 years	Primary endpoint = MACE, which included composite of cardiovascular death and a ventricular arrhythmia, terminated by the ICD. LGE presence HR 14.5 (6.06–32.61).
		Gulati et al. (25)	472	5.3 years	Primary endpoint = ACM, cardiac transplantation. LGE per 1% increment 1.11 (1.06–1.17).
		Masci et al. (26)	228	1.9 years	Primary endpoint = CV death, congestive heart failure, aborted SCD. LGE extent HR 5.104 (2.783–9.361).
		Perazzolo Marra et al. (29)	137	3 years	Primary endpoint = SCD, sustained ventricular arrhythmia, appropriate ICD intervention. LGE presence HR 4.17 (1.56–11.2).
		Puntmann et al. (44)	637	1.8 years	Primary endpoint = ACM. LGE presence HR 2.9 (1.4–6.3).
T1 Mapping	Research tool	Barison et al. (43)	89	2 years	Primary endpoint = composite of cardiovascular death, hospitalization for heart failure, and appropriate defibrillator intervention. ECV HR 8.59 × 107 (1,503–4.80 × 1,012).
		Puntmann et al. (44)	637	1.8 years	Primary endpoint = ACM. Native T1 HR 1.1 (1.06–1.15), ECV per % change HR 1.1(1.05–1.14).
		Nakamori et al. (45)	107	Retrospective events	Primary endpoint = ventricular arrhythmia. Native T1 each 10-ms increment OR 1.14 (1.03–1.25).
FT-CMR: LV strain	Research tool	Buss et al. (60)	210	5.3 years	Primary endpoint = combination of CV death, heart transplantation, and aborted SCD. GLS HR 1.33 (1.21–1.47), GCS HR 1.23 (1.13–1.34), GRS HR 0.89 (0.84–0.95).
		Romano et al. (65)	507	4.4 years	Primary endpoint = all-cause death. GLS HR 1.402 (1.299–1.513).

*LV, left ventricular; LVEF, left ventricular ejection fraction; HF, heart failure; LVEDVi, indexed left ventricular end diastolic volume; ACM, all-cause mortality; LV-EDV, left ventricular end diastolic volume; LVMi, indexed left ventricular mass; SCD, sudden cardiac death; CV, cardiovascular; RV, right ventricular; RVEF, right ventricular ejection fraction; TAPSE, tricuspid annular plane systolic excursion; RVEDVi, indexed right ventricular end diastolic volume; ICD, implantable cardioverter defibrillator; LA, left atrial; LAVi, indexed left atrial volume; LGE, late gadolinium enhancement; MACE, major adverse cardiac events; ECV, extracellular volume; FT-CMR, feature tracking-cardiac magnetic resonance imaging; GLS, global longitudinal strain; GCS, global circumferential strain; GRS, global radial strain*.

Much of the current CMR tools for characterization and predicting outcomes in DCM rely on multiple dedicated imaging sequences, followed by significant time devoted to qualitative post-processing in the evaluation of structure, function and tissue characterization. Despite their feasibility and utility, they are often not fully exploited in clinical practice due to these time constraints on clinical workflow. Even if employed, this often occurs *ad-hoc* and limited to one or two additional parameters evaluated in uniform manner, rather than assimilated in multi-parametric fashion for personalized characterization and risk stratification. Fully integrated analysis of all these features and metrics could aid better selection of patients who might benefit from earlier medical intervention, need closer surveillance regardless of LVEF, and those who we can more confidently discharge or halt medical therapies following improvement in their cardiac status (5, 13, 66).

### The Role of Genetics in DCM Characterization

It is increasingly appreciated that DCM has a genetic basis, with disease causing variants identified in up to 40% of families of DCM and 25% of presumed sporadic cases (67). Some of these genetic mutations can predispose carriers toward significant brady- or tachy-arrhythmias, or in the presence of environmental factors such as alcohol, can be the driver for a more severe phenotype, and there is a suggestion that a genetic basis could explain the higher prevalence of DCM seen in particular ethnic groups (67–69). There has been an expansion in the reported breadth of genes associated with the DCM phenotype, particularly in recent times with the arrival of next generation sequencing methodology (70, 71). However, robust genotype-phenotype correlations are not always feasible as the genes implicated encode proteins with a variety of different functional properties, making it challenging to harmonize the extent of genetic influence on the spectrum of structural and functional changes in those with DCM (71). Furthermore, it is challenging to clinically define and manage the large number of variants of uncertain significance (VUS), inadvertently arising as a result of the high throughput of current genetic testing.

Being able to discern the full scope of genetic influence in those with DCM will further help tease underlying drivers of disease manifestation and offer the opportunity to establish a deeper characterization of the phenotype. Coupled with the challenge to examine genetic influence on the spectrum of structural and functional changes, the addition of mutation status to clinical and imaging parameters may improve risk stratification and potential treatment strategies beyond the consensus management for heart failure with reduced ejection fraction (HFrEF) (72).

## AI Applications in the CMR Characterization of DCM

AI is the division of computer science that deals with the ability of computer systems to use algorithms in order to interpret and learn from data, and successfully perform tasks that would normally require human intellect and input. Over time, we have seen AI gaining popularity in medicine, having applications within medical record mining, predictive clinical application systems, virtual patient care and, its widest application, medical imaging (73, 74). In short, AI has the potential to perform routine tasks more efficiently or provide new insights into disease processes, that were previously not achievable by manual review and analysis due to time and labor constraints (75).

**Figure 4:**
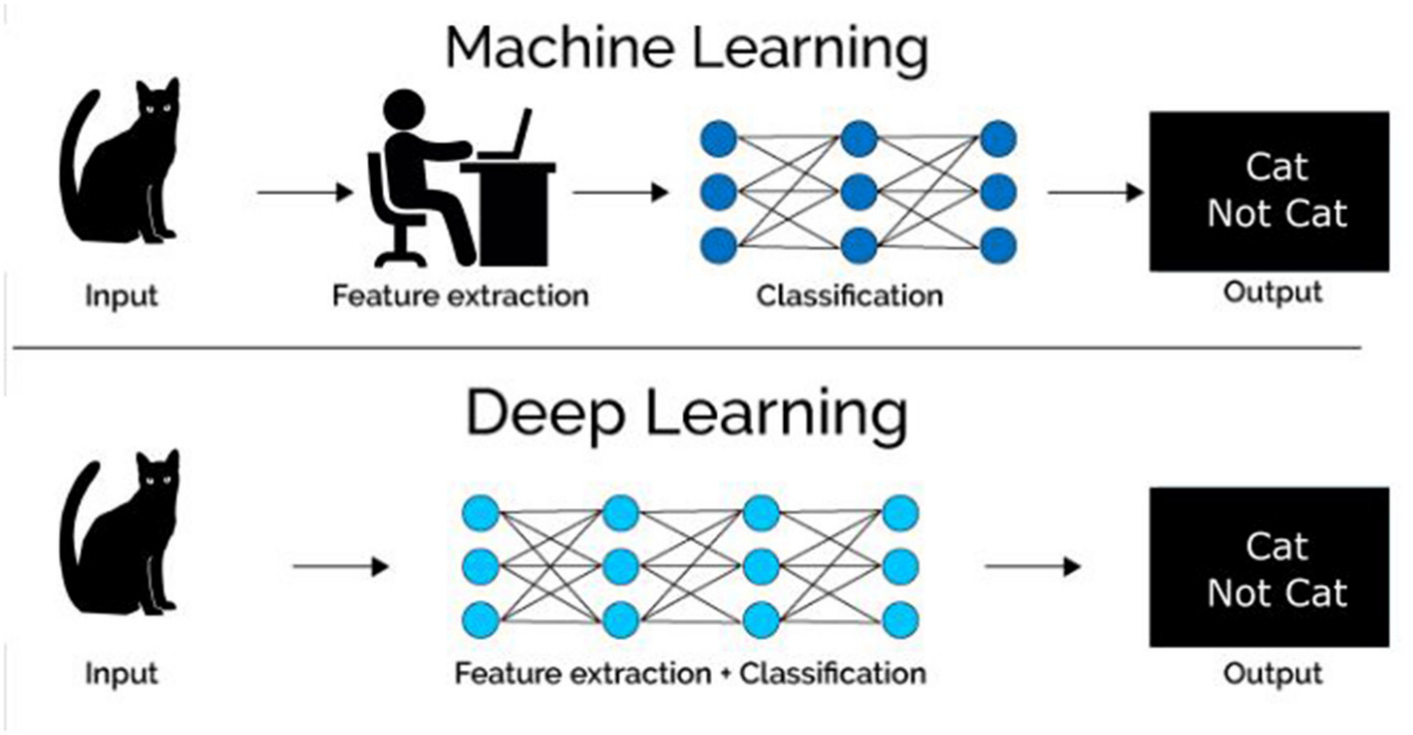
Visual example of the differences between machine learning (ML) and deep learning (DL) methods.

**Figure 5:**
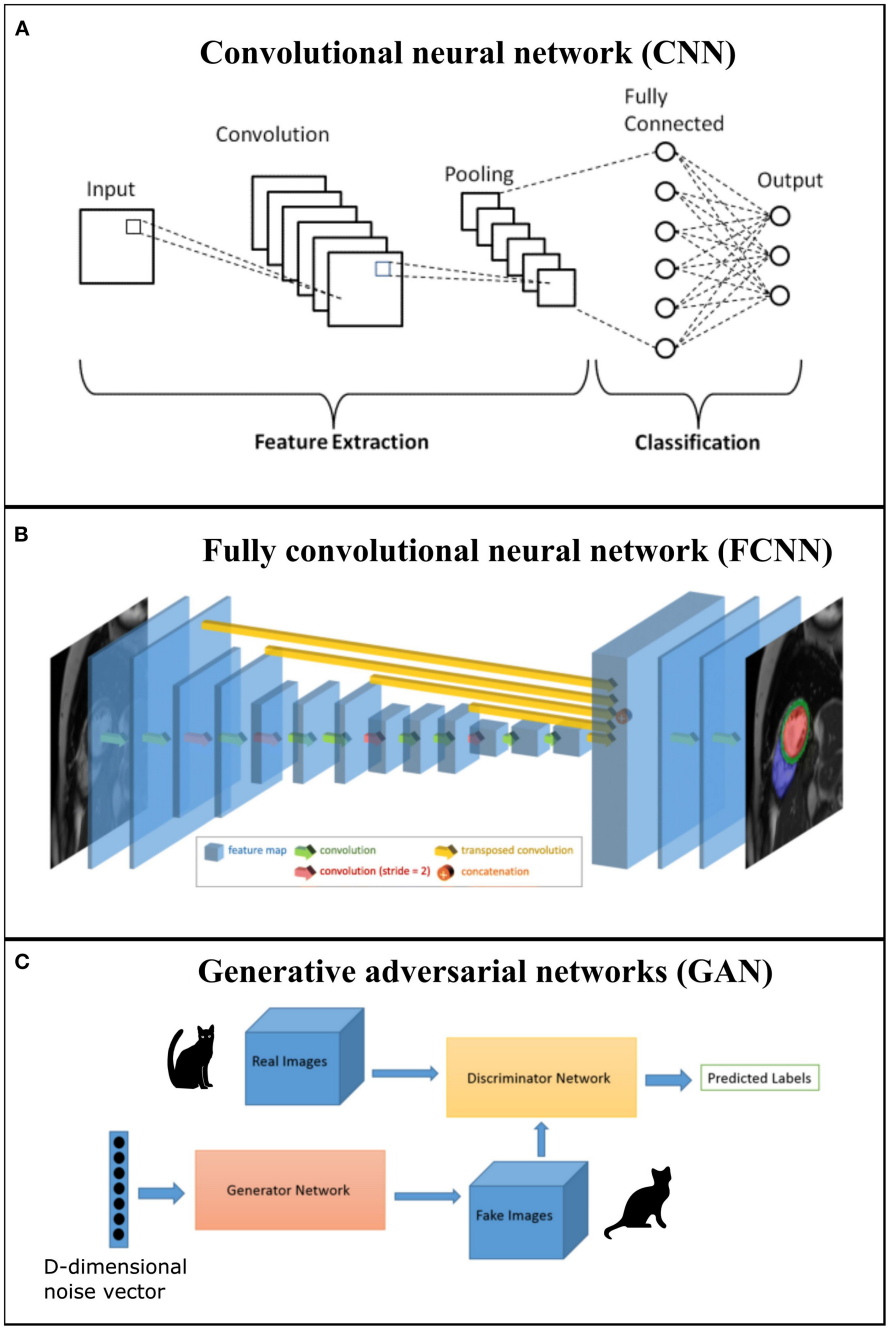
**(A)** Example of a convolution neural network (CNN) where first section corresponds to the feature extraction and second section to classification; **(B)** Example of a generic fully convolutional neural network (FCNN) with feature map volumes that are color-coded by size. Figure adapted from Bai et al. (78); **(C)** Example of a generative adversarial networks (GAN) that comprises two networks (generator and discriminator).

AI, machine learning (ML), and deep learning (DL) are three terms often used interchangeably but are essentially hierarchical. AI is the overarching concept aiming to develop computers with human intelligence. ML is the subfield of AI that gives computers the ability to learn without being explicitly programmed (76). DL is a subset of ML algorithms called neural networks. Neural networks are algorithms that mimic the human brain's behavior in decision-making and try to find the most optimal path to a solution. Traditionally, ML methods contain a feature engineering phase, where experts propose a set of hand-crafted features to facilitate the learning from examples. This phase is very important and affects the overall performance of the learning system. In a DL pipeline, feature extraction is embedded in the learning algorithm where features are extracted in a fully automated way and without any intervention of a human expert (see Figure 4 for visual example of the ML and DL method). A number of fundamental neural network architectures lie at the basis of DL models, and we provide a basic introduction to their concepts. However, for a more comprehensive overview of these architectures and DL algorithms for cardiac image segmentation, we refer the interested reader to Chen et al. (77), Convolutional neural networks (CNN) are the most popular class of DL network, widely applied in CMR, utilizing a patch-based image extraction approach (see Figure 5A for an example of a CNN network). As opposed to this conventional neural network, a fully convolutional neural network (FCNN) performs more efficient and accurate pixelwise segmentation by leveraging upsampling layers to concatenate multi-scale features obtained through a series of convolutions applied to the entire image (see Figure 5B for an example of a FCNN) (77). Finally, another emerging class of DL algorithms are the Generative adversarial networks (GAN). These consist of a pair of neural networks, contesting one against another (“adversarial”), in order to generate new, synthetic instances of data that can pass for real data (see Figure 5C for an example of GAN architecture).

There are three important types of AI algorithms: (1) Supervised learning algorithms try to model relationships and dependencies between the target prediction output and the input features or observations such that we can predict the output values for new data based on the learnt relationships. This is the most partial and widely adopted form of AI, but it requires a large amount of labeled training datasets; (2) Unsupervised learning is where there are no corresponding output variables, and the goal is to discover relationships between the input features or reveal the latent variables behind the observations; and (3) Reinforcement learning aims to learn a mapping from situations to actions so as to maximize a scalar reward or reinforcement signal. A key difference with supervised learning is that the reinforcement learning agent is never told the optimal action, instead it receives an evaluation signal indicating the goodness of fit for the selected action.

Medical image analysis involves the use of images generated in clinical practice, that can be interpreted to improve our ability to solve clinical problems and make treatment decisions more effective (79). As the increasing wealth of digital data becomes more accessible, clinicians need to be able to find more efficient ways of meaningfully combining this data to boost precision-based healthcare.

Due to the spatial and temporal pathologic heterogeneity of particular clinical phenotypes, such as DCM, the ability to accurately identify and extract relevant imaging biomarkers in routine clinical practice is prone to subjective errors and has low reproducibility when carried out by hand. Over the last decade, AI has made significant progress in the field of medical imaging, improving techniques involved in acquisition, analysis, and interpretation with gradually less human oversight involved in the process (74). These efforts reduce the variability associated with subjective image interpretation, and moreover, enable feature extraction around regions of interest. It has the potential to provide quantifiable features that relate more objectively and in more detail with relevant clinical information (80).

### Overcoming the Barriers to Fully Automated Image Segmentation

The opening act to CMR characterization involves image acquisition and segmentation, prior to feature extraction. The quality of this step is essential to the outcome of further downstream analysis and provides baseline cardiac parameters as well as the untapped features that might better describe cardiac function in specific cohorts. Assessment of the anatomical features following cine acquisition includes assessment of myocardium, pericardium, all 4 cardiac chambers, valves and vascular connections. Typically, a visual quality assessment is needed first to ensure the signal-to-noise ratio is enhanced by adequate positioning and breath-holding technique, limited blurring by cardiac gating, and appropriate planning for each subsequent image plane acquisition. Following the anatomical review, long and short axis cines are acquired that enable dynamic views of the global heart function. Segmentation by manual planimetry, or by semi-automated methods with clinician oversight enables the reproducible 3-dimensional (3D) assessment of atrial and ventricular volumes, LV mass and EF quantification.

Studies have shown that DL methods can outperform conventional ML, and in some cases, even better in both detection and segmentation tasks analyzed by human expertise (80, 81). CNNs are the technique of choice and the most successful type of models for image analysis (82, 83). Efficacy of the DL models is often assessed in the form of pixel classification accuracy. Although different methods for assessing this exist, the preferred evaluation metric for DL-based segmentation approaches is the Dice metric, which evaluates the overlap between automated segmentation and the ground truth segmentation. The Dice metric has values between 0 and 100%, where 0 denotes no overlap and 100% denotes perfect agreement.

One of the main challenges to implementation of CNNs in medical imaging is the lack of high-quality expert annotated data, available for training the DL network. Furthermore, these datasets often suffer from class imbalances due to certain conditions being encountered less frequently, thereby making it more difficult for a CNN to generalize and limiting large scale CMR evaluations. As highlighted in Table 2, whilst the segmentation performance of state-of-the-art DL methods is commended, it is evident that the number of DCM cases encountered in these evaluations has significantly low representation. Given the heterogeneity of this condition with many individuals at presentation subject to highly remodeled ventricles and rotated cardiac axes, these current automated segmentation methods may not yet be robust enough for the deployment and evaluation of this phenotype.

**Table 2:** State of the art DL architecture on CMR datasets and number of DCM cases encountered in test datasets.

**Selected Work, References**	**DL Architecture and type of images**	**Structures Segmented**	**No. subjects in total used for training/validation/testing**	**Dice metric between AS and MS: LV cavity**	**Dice metric between AS and MS: LV myocardium**	**Dice metric between AS and MS: RV cavity**	**No. of DCM test cases**
Bai et al. (78)	2D FCNN, SAX images	Biventricular and atria	Training 3,975 Validation 300 Testing 600	Mean 0.94 (SD 0.04)	Mean 0.88 (SD 0.03)	Mean 0.90 (SD 0.05)	142
Tran (84)	2D FCNN with transfer training, SAX images	Biventricular	Training 131 Validation 100 Testing 115 (LV), 32 (RV)	Mean 0.92 (SD 0.03)	Mean 0.96 (SD 0.01)	Mean 0.84 (SD 0.21)	Unspecified; mix of cardiac conditions
Isensee et al. (85)	Ensemble FCNN (2D and 3D U-net), SAX images over full cardiac cycle	Biventricular	Training 100 Testing 50	Mean 0.945	Mean 0.905	Mean 0.908	10
Tao et al. (86)	2D FCNN, SAX images from multivendor dataset	LV/Myocardium	Training 400 Testing 196	Mean 0.92 (SD 0.06)	Mean 0.94 (SD 0.05)		46
Khened et al. (87)	2D Densenet (FCNN), SAX images	Biventricular	Training 700 Validation 300 Testing 490	Mean 0.93 (SD 0.05)	Mean 0.89 (SD 0.03)	Mean 0.91 (SD 0.05)	10
Jang et al. (88)	2D M-net (FCNN), weighted cross entropy loss, SAX images	Biventricular	Training 80 Testing 20	Mean 0.938 (SD 0.05)	Mean 0.879 (SD 0.04)	Mean 0.890 (SD 0.07)	10
Fahmy et al. (89)	2D FCNN with alignment and T1 estimation, SAX images	LV/Myocardium	Training 63 Testing 147		Mean 0.85 (SD 0.07)		Unspecified; mix of cardiac conditions
Avendi et al. (90)	2D CNN for localizing LV, stacked autoencoders for shape inference. Deformable model for segmentation, SAX images	LV	Training 45 Validation 30 Testing 30	Mean 0.94 (SD 0.02)			Unspecified; mix of cardiac conditions
Avendi et al. (91)	2D CNN for localizing RV, stacked autoencoder for automatic initialization. Deformable model for segmentation.	RV	Training 16 Testing 16			Mean 0.83 (SD 0.14)	Unspecified; mix of cardiac conditions from dataset of 48 patients
Oktay et al. (92)	2D FCNN with anatomical shape priors, SAX images	LV/Myocardium	Training 900 Validation 100 Testing 200	Mean 0.939 (SD 0.02)	Mean 0.81 (SD 0.03)		0

*DL, deep learning; AS, automated segmentation; MS, manual segmentation; LV, left ventricle; RV, right ventricle; FCNN, fully convolutional neural network; CNN, convolutional neural network; SAX, short axis*.

To ensure any of these methods can translate into clinically useful tools in the evaluation of DCM, it is essential they are complemented by high quality datasets, that help improve the accuracy of segmentation and classification tasks, whilst providing large variability in terms of the clinical phenotype and image acquisition modules, thus enabling generalizability (87, 91). However, manual annotations of large datasets that are able to encompass this scale of heterogeneity is no easy feat, being costly and requiring extensive expert time for good quality annotation. This could be partly overcome with data sharing initiatives and collaborations between CMR centers to obtain large repositories of images with associated clinical information. This is not inevitably a seamless solution, as there are often ethical and legal requirements to satisfy within all participating sites, with limits set on how and where specific data can be utilized during the development and deployment of the pipeline.

Encouragingly, over the years open technical challenges and several publicly available datasets have been made available, helping to unravel this generalizability issue (93, 94). The UK Biobank (UKBB), although limited to a single CMR vendor, provides one of the largest imaging datasets facilitating the exploration of DL capabilities in a large general population whilst solving issues relating to ethics and clinical data aggregation. This was harnessed recently in a genome-wide association study of CMR-derived LV measurements in ~36,000 participants from the UKBB to study the relationship between genetic variants associated with LV structure and function, and risk of incident DCM (95).

Another way of improving the generalizability during training and take advantage of the limited amount of high-quality labeled data is the strategy of data augmentation. It is possible to artificially increase the variation of examples encountered by applying random transformations such as image rotation by certain degrees, image scaling to increase variations in organ size, changing image orientation with random horizontal or vertical flips and even inclusion of random “noise” to images (94, 96, 97). Whilst this option effectively enables the acquisition of more labeled data, the diversity in practice may still be limited in terms of reflecting the full spectrum of the DCM phenotype and the pixel-level differences of images obtained from different CMR vendors. The breakthrough in improving this generalization of networks to reliably segment heterogenous phenotypes acquired from different CMR vendors and clinical sites was demonstrated recently by Chen et al. (94). Unlike the efforts to solely re-train or fine tune networks to improve the performance on a specific dataset, they explored the pre-processing step of data normalization enabling their network to deal with the distribution changes amongst input features from multi-source images. This overcomes the small differences in features arising from images obtained from different scanners and the overfitting to distribution changes that occurs with network development from a single source. Along with data augmentation strategies, their approach achieved encouraging results in terms of reliable segmentation accuracy across test images from multi-scanner and site domains (mean Dice metric of 0.91 for the left ventricle, 0.81 for the myocardium, and 0.82 for the right ventricle from a single site dataset; and 0.89 for the left ventricle, 0.83 for the myocardium from a multi-site dataset).

### DL-Based Global Assessment of Function

Recent DL techniques have enabled rapid expansion in the CMR domain to achieve robust contour identification and accurate classification performance, whilst significantly minimizing the extent of post-processing involved in volumetric data calculations (98). Emerging approaches that have recently become commercially available, have further demonstrated the feasibility and precision of fully automated dynamic measurement of LV volumes (99, 100). Utilizing anatomical localization methods to determine relevant boundaries between structures, contours are created in consecutive frames of the cardiac cycle with LV volume/time curves derived at no extra time expense and with high correlation to the manual reference technique (99). Whilst such applications show promise for the application to evaluating DCM cohorts, the development and training of this technique has been based on very limited representation from such patients, with less accuracy seen in those with significantly dilated ventricles and whose impaired breath-holding technique can lead to significantly more artifacts, reducing image quality. Furthermore, the details of the DL pipeline are not disclosed by the manufacturer and this lack of transparency will make it difficult to optimize the current algorithm in order to generalize to other scanners and more diverse patient cohorts.

Motivated by these limitations, Ruijsink et al. (17) developed a robust, accurate and fully automated framework for CMR cardiac function analysis which included comprehensive quality control detection using a CNN to limit erroneous output. Segmentation of both ventricles in all frames was then executed utilizing a 17-layer 2D-FCNN, prior to an iterative alignment process to correct for any differences in breath-holding and motion. After validating their framework that presented with high correlation to manual analysis, biventricular volume curves were generated for over 2,000 healthy individuals to obtain a more detailed description of cardiac function, inclusive of diastolic parameters such as peak early filling rate, atrial contribution, and peak atrial filling rate. These parameters stratified healthy patients by age categories, with lower filling rates correlating with older age–a relationship consistent with the known increase in ventricular stiffness with age (101). Considering that these LV filling patterns also appear capable of distinguishing the different categories of diastolic dysfunction characterized on echocardiography, it is anticipated that this method could enable within DCM subgroups detection of those with persistent diastolic impairment despite LV systolic recovery on medical therapy, and identify patients with subclinical disease who will require closer surveillance (102). These parameters are feasible with no additional imaging outside routine care and can occur at no extra time-cost whilst the routine clinical analysis is ongoing. In terms of the potential clinical application to evaluation of the DCM phenotype, it is significant that the method employed by Ruijsink et al. (17) performed similarly well in unseen patients with cardiomyopathy as well as those without cardiac disease. It has been reported from studies on emerging 4D flow CMR, that DCM patients have altered and heterogenous diastolic flow patterns that occur due to abnormal filling kinetics and varying degrees of pathological geometrical configuration of the LV (103). This highlights the potential role offered by fully automated LV filling assessment in differentiating those with persistent altered filling patterns and abnormal diastolic flow, thereby remaining at risk of relapse compared to those who have truly achieved recovery and remission. Based on this promising AI tool, current work by this group is also exploring the innovative use of GANs to generate realistic CMR images from any domain in order to advance the generalization of the network and robustly deal with clinical CMR data from multiple centers, vendors, and field strengths (104). Given the feasibility to evaluate both ventricular filling profiles, and the suggested prognostic role of serial revaluation of RV function in the follow up of DCM, the characteristics and clinical utility of RV filling patterns over time will be another area of application in the DCM population (105).

### DL-Based Tissue Characterization

State-of-the-art algorithms utilized in scar segmentation are commonly semi-automated, fixed-model approaches where the pixel intensities of scar regions are exploited through a process of thresholding (106). This requires a user-selected area of interest and knowledge of the nearby intensities of healthy nulled myocardium, prior to operating a region growing process to segment the scar region. These methods are currently popular for segmenting contiguous regions of scar, and are highlighted for their reproducibility, with encouraging performance against consensus segmentation by experts (106). Whilst simple to implement, they remain heavily user dependent for pre-processing with respect to definition of the myocardial borders, activating the boundaries of interest, initialization for region growing in each slice, and the subjective baseline selection of remote healthy myocardium as well as the perceived extent of scar. More automated approaches have been developed to help minimize the degree of user interaction whilst maintaining reproducible performance (106). These methods mostly utilize clustering techniques to fit data of different tissue signal intensities in order to characterize the voxels belonging to scar regions (106). Whilst they show good correlation with the fixed-model approaches in accurately identifying LGE, they unfortunately are not robust enough for clinical translation due to failure to accurately segment scar where CMR-LGE images are affected by noise or share homogenous signal intensity distributions within myocardial boundaries and other nearby tissues (106–109). This limitation is particularly important as most of these traditional methods have been validated on CMR-LGE images obtained from patients with coronary disease, where the pattern of scar is subendocardial as opposed to that seen, if present, in DCM and other non-ischaemic cohorts, occurring in the mid to epicardial wall segments, where tissue intensity homogeneities are more likely to be encountered.

As the attention of CMR segmentation transitions toward more DL-based approaches, it is hoped that these innovative techniques will also facilitate a more practical and reliable means of achieving standardized quantification of LGE. This is highly desirable, given the suggestion that even after adjustment of LVEF, the proportion of LGE assists the clinical stratification of DCM patients who are prone to a higher risk of death and hospitalization (28). Not to mention, the ability to efficiently characterize border zone tissue–areas of variable transition between scar and normal tissue that have arrhythmogenic potential, thereby helping to identify those at risk of malignant arrhythmias and more likely to benefit from ICDs (110).

A successful FCNN architecture, the ENet was recently harnessed to deal with the task of scar segmentation (111). Several variants of this popular architecture have been adapted to enhance the accuracy of different cardiac segmentation tasks (see Table 2). However, the pursuit of scar segmentation is a relatively new concept. In this work by Moccia et al. (111), the ENet was adapted and evaluated to see if pre-identified LV regions could enable more accurate scar segmentation than current methods, and furthermore whether a fully automated output of scar segmentation was feasible and maintained a similar or improved accuracy. As a proof of concept, this method showed both protocols were able to identify scar on the CMR-LGE images without the need for pre-processing extraction steps. However, it was the semi-automated method with a priori knowledge of the restricted myocardial boundary in which to search for scar, that outperformed state of the art CMR-LGE segmentation algorithms and was closest to expert annotation (with a sensitivity of 0.88 and Dice coefficient of 0.71). This is still an important breakthrough, holding advantages over current efforts to quantify scar by minimizing subjective evaluation, user interaction and any parameter tuning prior to implementation. Expanding the training datasets to incorporate the variability of scar seen in those with a sole DCM phenotype and those with accompanying embolic sub-endocardial scar, could be an encouraging start to help encode the high variability of scar dimensions seen in this population. This study provides an important step forward in the clinical practice of scar quantification, and by enhancing the pixel classification through training, the ENet would not only acquire improved segmentation performance, but would be more generalizable to the DCM population.

In conjunction with acquiring diverse DCM datasets, image data augmentation is another common method to artificially boost training datasets in order to improve performance and generalizability of a deep learning model. This may be particularly relevant with regards to the DCM population, where a disconnect exists between high demand for sufficient training images and the variability of scar presence across the spectrum of patients, in essence, limiting the real-world availability of training examples (24). This was recently explored in the simulation of scar tissue on the LGE-CMR images of healthy patients (112). Lau et al. (112) utilized their GAN framework which could additionally incorporate domain-specific knowledge, to simulate various scar tissue shapes in different positions. These images were highly realistic as demonstrated by the improved segmentation prediction of scar tissue pixels correctly identified during testing from 75.9 to 80.5% and the qualitative assessment that imaging experts were unable to reliably distinguish between simulated and authentic scar.

A stream of work has focused on extending the use of a trained FCNN to assist analysis of myocardial tissue characterization by means of automated native T1 mapping (89). With good agreement to manual calculations, this showed promise for an automated pipeline to minimize the workflow involved in quantifying global T1 characteristics. This was validated on a single scanner, with further study needed to see if this method can be applied to other mapping sequences such as the modified Look-Locker inversion recovery (MOLLI) or the contemporary shortened modified Look-Locker inversion (shMOLLI) method, that is more acceptable and compatible with typical limits for end-expiration breath-holding in patients (113). Puyol-Antón et al. (114) evaluated an automated framework for tissue characterization using the shMOLLI method at 1.5 Tesla using a Probabilistic Hierarchical Segmentation (PHiSeg) network. This method models the probability distribution of pixel-wise segmentation samples from the input image and generates an uncertainty map to quantify the degree of error in segmentation, so that erroneous representations are not utilized for T1 mapping. A morphological operation was then applied to detect the LV-RV intersection and delineate LV free wall from the interventricular septum. T1 ranges were obtained from the uncovered myocardial regions of interest with correction for T1 from the ventricular blood pools to improve discrimination between healthy subjects and those with cardiovascular disease (115). Using this proposed method, they characterized global and regional T1 values from over 10,000 subjects from the UKBB dataset which included a significant proportion of non-ischaemic cardiomyopathies. In line with present comprehension, they demonstrated that for those conditions in which diffuse fibrosis is more prevalent such as DCM, hypertrophic cardiomyopathy (HCM), and cardiac sarcoidosis, they found significantly higher T1 values (all *p* < 0.05). The quality control process is an important feature for clinical scalability of this tool and would enable this supplementary prognostic information to be added to each DCM case in a uniform manner with no added time-expense. Furthermore, it would enable large scale application to assess the role that native T1 analysis may have in deriving enhanced prediction of adverse outcomes for particular subgroups of DCM, particularly those who remain at risk despite having only mild or moderately impaired LV function. In order to be more generalizable, the proposed model requires validation on datasets acquired from the various different vendors available currently in clinical practice.

## Clinical Risk Prediction and the Role for AI

Whilst clinical risk prediction models for prognostic assessment exists for heart failure populations in general, these tend to be below par when utilized in DCM patients (116). For the most part, these models are derived mostly from heart failure due to ischaemic etiology, which on average is associated with a higher mortality risk, and tends to affect older individuals who have other associated cardiovascular risk factors (116). DCM tends to affect younger patients with the vast majority having mild dysfunction remaining stable for many years. Alternatively, they can also be characterized by incidences of sudden progressive dysfunction, or by those without severe LV dysfunction who remain susceptible to ventricular arrhythmias and sudden cardiac death; both of which would not be accounted for by conventional risk prediction models (1, 117, 118).

Emerging techniques in AI pertaining to the exploration of informative clinical biomarkers potentially offers a better appreciation of the phenotypic heterogeneity underlying DCM, with refined clinical implications in risk stratification, earlier detection and personalized treatment strategies (116). In this section, we provide an overview of these AI-based clinical applications that are primed to advance the field of clinical risk prediction in DCM.

Chen et al. (119) recently evaluated their ML model based on 32 features obtained from baseline patient characteristics, bloods, ECG, echocardiography and CMR, and assessed its performance in predicting cardiovascular events in a group of severe DCM patients. Feature selection occurred with Information Gain, an attribute selection technique that enables rapid classification of the most relevant features to the cardiovascular events. Although a number of ML models performed well in terms of accuracy and ability to discriminate between an event and non-event for each feature, a naïve Bayes classifier was selected as the model of choice due to the additional transparency offered with the generation of conditional probabilities associated with each outcome. This was the most meaningful in terms of clinical translation, as the relevant significant features could form part of the clinician's probabilistic reasoning in the decision aid for guiding a patient's treatment. This would need further exploration in subsequent iterations of the model and prospective clinical trials in order to evaluate the capability to assign risk to particular patients. Nonetheless, by handling most of these features that are often used variably in practice for risk prediction such as LGE extent, degree of mitral regurgitation, and QRS duration, this model outperformed current scoring systems and LVEF alone for the prediction of cardiovascular events in each patient [AUC, 0.887 (95% confidence interval, 0.813–0.961)].

By integrating longitudinal clinical, biochemical and echocardiography imaging data from over 4,000 patients with cancer, Zhou et al. (120) built predictive supervised ML models for applicable cardiovascular outcomes such as heart failure and *de novo* cancer therapy-related cardiac dysfunction (CTRCD). Based on a number of model iterations from five different classification methods, logistic regression provided the optimal classification performance, with an area under the receiver operating characteristic curve of 0.882 (95% CI, 0.878–0.887) for heart failure and 0.802 (95% CI, 0.797–0.807) for *de novo* CTRCD. They identified a combination of 9 clinically relevant variables that were strong predictors for these outcomes (*p* < 0.05) and maintained this high performance even when tested on data from separate time points to the training dataset. As one of the potentially reversible causes of DCM, this generalizability and high performance in predicting CTRCD over time makes ML models such as this a potentially promising tool for real-world cardiac risk assessment in cancer patients throughout their treatment journey. As these models are evaluated in larger cohorts with fine-tuning and model-specific variable selection to enhance performance, this group are also collaborating with clinicians to develop integrated risk calculators with outcomes in order to test the prospective potential of ML-derived biomarkers in cardio-oncology practice.

Treatment of DCM is predominantly as part of the management of heart failure with reduced ejection fraction. This is directed at reversal of adverse LV adaptive mechanisms that occur in progressive LV dysfunction, so called LV reverse remodeling (LVRR), and is a key determinant of prognosis in DCM (1). Up to 40% of patients are reported to experience this within two years, due to removal of the precipitating factor or induced through medical therapies and/or cardiac resynchronization therapy (CRT) in those who have left bundle branch block and subsequent dyssynchronous ventricular activation (1, 121). Beyond medical therapy, CRT in this setting has clear efficacy in terms of improving symptoms and reducing mortality (122). However, determining those who will “respond” to this therapy moving toward and maintaining remission in the long-term, as opposed to those who may be non-responders, is still a current challenge in the clinical setting (1, 123). Whilst multiple clinical, imaging, and even device implant factors are associated with the likelihood of positive response to CRT, gaps of knowledge still remain regarding timing of this evaluation and how to leverage this information to identify evidence of early remodeling (121, 123).

A number of ML algorithms have explored the combined assessment of different clinical variables in predicting response to CRT and recovery of myocardial function. Multiple kernel learning (MKL) has been used by different groups as it offers the possibility of combining data from different sources as different kernel matrices, and it learns the importance of each kernel. For example, a framework was developed by Peressutti et al. (124), which captured LV motion information from spatio-temporal atlases deployed in CMR imaging from a mixed cardiomyopathy cohort. They then applied a supervised MKL to combine and evaluate the relationship between the rich motion descriptors and selected clinical information derived from clinical reports, ECG and data from echocardiography. Although applied to a limited cohort of 34 patients selected for CRT, this coupling of electro-mechanical LV data to clinical metrics achieved an accuracy of 94% in predicting super-responders and 91% for non-responders, at 6 months post CRT implant. Future work incorporating anatomical descriptors into the atlases could potentially inform of mechanistic differences between responders and non-responders.

Cikes et al. (125) utilized an unsupervised MKL algorithm in a heart failure cohort of over 100 patients recruited from the Multicenter Automatic Defibrillator Implantation Trial with Cardiac Resynchronization Therapy (MADIT-CRT trial). This trial had previously demonstrated the added benefits CRT added to ICD in terms of decreased risk of heart failure events in those with a low LVEF and wide QRS duration on ECG (126). In order to provide meaningful classification of this phenotypically heterogenous cohort, this algorithm was used to cluster patients by clinical characteristics, biochemical biomarkers, ECG, and echocardiography-derived patterns. They observed specific phenogroups with characteristics predictive of best volume reduction, CRT response, and overall better treatment effect on heart failure outcomes [hazard ratio (HR) 0.35; 95% confidence interval (CI) 0.19–0.64; *P* = 0.0005 and HR 0.36; 95% CI 0.19–0.68; *P* = 0.001].

Although most of the examples above come from more diverse heart failure cohorts, ML within these settings clearly has the potential for novel integration of the readily available and extensive clinical, biochemical, and imaging parameters to phenotype heterogenous diseases, such as DCM. They offer the added advantage of exploiting this information as biomarker data to unearth and compare the similarities between subgroups, and importantly provide a degree of interpretability for the associations identified (125). Understanding the value and accuracy of this output is not only relevant to understanding how to improve the ML algorithm's operation, but is fundamental for bridging the gap to advances in the clinical application of these tools. This has been a need particularly for DL algorithms, which have made impressive leaps in performance and accuracy in some image classification tasks, but are often depicted as “black boxes,” offering little understanding to the prediction of their results.

Puyol-Antón et al. (127) offered in the first of its kind, an interpretable approach to a DL model for the prediction of CRT response. This framework was based around a DL-based generative model known as a variational autoencoder (VAE) which encodes the segmented biventricular data into a low dimensional latent space, followed by a primary task classifier of predicting those who would respond to CRT utilizing pre-treatment CMR images. A secondary classifier which follows a similar structure to the first, and then incorporates clinical domain knowledge to provide an explanatory concept within the encoded space. By example, they utilized the concept of septal flash–an identified pattern of early septal contraction and a marker of interventricular dyssynchrony (128). The classifiers enabled the separation of CRT responders and non-responders in the image domain with visualization of where the learned features of CRT responders corresponded to the clinical domain knowledge. This has important implications beyond predicting CRT response in DCM, with the potential ability of DL models to explore multiple validated clinical parameters involved in arrhythmia prediction and reverse remodeling as explanatory concepts, thus granting a better understanding of the disease process pathways and the varying responses of different subgroups (129).

## AI Tools for Integrated Imaging-Genetics in DCM

Supervised ML approaches improved the prediction of DCM patients mostly likely to experience LV reverse remodeling following the novel therapeutic approach of immunoadsorption and immunoglobulin substitution (130). The integration of overlapping myocardial gene expression patterns, using a support vector machine and random forest analysis, enabled the development of a robust classifier that helped distinguish responders to therapy, and enhanced predictions beyond clinical parameters and antibody response levels alone [sensitivity of 100% (95% CI 85.8–100%); specificity up to 100% (95% CI 79.4–100%); cut-off value: −0.28].

Similarly, Schmitz et al. (131) demonstrated that ML algorithms could be applied to identify predictive combinations of clinical and genetic markers that could enhance the classification of heart failure patients likely to respond to CRT treatment. This work proposed the concept of underlying genetic substrates that may exclusively or through interaction with other factors contribute to the remodeling phenotype of certain heart failure cohorts. This additional predictive information may provide some understanding of the variable responses to CRT therapy and help improve outcomes.

High fidelity ML models incorporating genetic sequencing, 2D and 3D CMR, explored the complexities surrounding the molecular mechanisms of DCM pathogenesis, mediated by titin-truncating variants (TTN) (132). These variants frequently associate with the DCM phenotype in sporadic and familial forms, and are also reported to occur in just under 1% of the general population, where their clinical significance is less clear (133, 134). However, following mass univariate analyses in healthy individuals, integrating multiple cardiac parameters obtained through CMR imaging, anthropometric variables and their relationship to detailed sequencing of the TTN genotype, Schafer et al. (132) demonstrated association between TTN status and higher LV volume due to eccentric remodeling. In leveraging this high-resolution phenotyping, this study highlighted the feasibility and benefits of ML in estimating the effect size of candidate pathogenic mutations on multiple metrics of cardiac morphology and function that are applicable to a deeper characterization of the DCM phenotypic spectrum. Furthermore, such studies are needed to help define the clinical indicators of an inherited DCM and the mechanistic interactions between genetic variants and other conditions that share some clinical features such as peripartum cardiomyopathy (135).

The potential of AI for rapid, purposeful extraction of high-quality imaging-derived phenotypes assimilated with genetics is also a promising arena for DL methods. Following rapid LV analysis in ~17,000 individuals by a FCNN highly optimized to automatic segmentation, the largest genome wide association study (GWAS) of image-derived phenotypes identified 14 significant loci for different LV traits that related to cardiac morphogenesis and risk of heart failure development (136). Furthermore, there were distinct loci that associated with LV remodeling, and others that were causal genes for multiple LV traits such as BAG 3 and TTN; two genes that also share implications in the pathogenesis of DCM. These findings emphasize a potential genetic basis underlining many of the structural and functional LV imaging traits routinely acquired through CMR imaging. With the unparalleled performance of fully automated imaging analysis by DL, it may be feasible to integrate this information and enrich our understanding of the pathogenic evolution of heart failure syndromes occurring in some DCM subtypes.

These promising applications highlight the unrivaled capability of AI to integrate complex structural, functional and genetic characteristics of DCM to better understand and characterize the phenotype. However, in order to universally translate to the clinical setting, they warrant validation and replication across the spectrum of LV dysfunction in order to tease the different pathways that are involved in the evolution of DCM.

## Future Perspectives and Conclusions

Advances in the applications of AI based medical innovations are rapidly increasing with a particular surge of interest within the specialties of Radiology and Cardiology (137, 138). Even now, across Europe and America, a number of innovations have already received Conformité Européenne (CE) marked or Food and Drug Administration (FDA) approval for the introduction of AI based solutions to simplify detection of cardiovascular risk and enable efficient, personalized disease prediction across a range of imaging modalities and clinical platforms (137, 138).

Despite this rapidly evolving landscape for AI opportunities in cardiac healthcare, there are still some limitations that need to be addressed before such applications can be successfully deployed into clinical practice. Firstly, the generalization of the methods, as most are only validated with high-quality data from standardized research environments which don't necessarily generalize well to external databases. To overcome this limitation, we think that AI models need to be validated in external databases that reflect real-world, heterogenous populations, and tested using decentralized techniques such as federated learning in order for them to be relevant and personalized to specific cohorts. A pioneer example of such initiative is the partnership between the British Heart Foundation and the Health Data Research UK (HDRUK), enabling access to the UK's large-scale and diverse cardiovascular data resource, where population-wide data analysis can be utilized to extract valuable information from unstructured data and investigate novel insights into cardiac disease pathways.

Another well-known pitfall of AI models is that they are “black boxes,” being difficult to gauge how they reach their output decisions and predictions. Explainable AI is a new branch of AI that aims to add interpretability to the models. From our point of view, this is likely to facilitate faster adoption of AI systems into the clinical healthcare setting and will help foster vital transparency and trust with their users.

For the DCM population, this further research from AI tools is welcomed and needed to find meaningful insights that are able to enhance the rapid, reliable automation of all relevant imaging indices for characterizing the phenotype. If these could help define the relationships between imaging phenotypes, genomic features and the impact of specific precipitant factors, then it may be possible to generate biomarker profiles to discover clusters of DCM patients that have similar outcomes, to better understand their similarities and furthermore, understand the influence of different treatment strategies. These biomarker indicators would be important in redefining risk stratification beyond LVEF, enabling a multi-parametric approach that can feasibly assess dynamic changes in cardiac status and help tailor treatments to the needs of a specific subtype and more specifically, the individual.

## Author Contributions

CA, EP-A, BR, RR, AC, and GC-W conceived and designed the work. CA and EP-A searched and read the CMR and AI literature, and drafted the manuscript with support from MR and BR. AC, RR, and GC-W provided the critical revision with valuable feedback to improve the manuscript. All authors read and approved the final version of the manuscript.

## Funding

This research was funded in whole, or in part by the Wellcome Trust/EPSRC Center for Medical Engineering at Kings College London (WT 203148/Z/16/Z). EP-A was supported by the EPSRC (EP/R005516/1, EP/P001009/1) and by core funding from the Wellcome/EPSRC Center for Medical Engineering (WT203148/Z/16/Z). BR was supported by the NIHR Cardiovascular MedTech Co-operative award to the Guy's and St Thomas' NHS Foundation Trust. CA and MR are supported by the UKRI London Medical Imaging and Artificial Intelligence Center for Value Based Healthcare (RE15376).

## Conflict of Interest

The authors declare that the research was conducted in the absence of any commercial or financial relationships that could be construed as a potential conflict of interest.

## Publisher's Note

All claims expressed in this article are solely those of the authors and do not necessarily represent those of their affiliated organizations, or those of the publisher, the editors and the reviewers. Any product that may be evaluated in this article, or claim that may be made by its manufacturer, is not guaranteed or endorsed by the publisher.
